# Magnitude of Rotavirus A and Campylobacter jejuni infections in children with diarrhea in Twin cities of Rawalpindi and Islamabad, Pakistan

**DOI:** 10.1186/s12879-019-4575-1

**Published:** 2019-11-21

**Authors:** Asma Sadiq, Habib Bokhari, Zobia Noreen, Rai Muhammad Asghar, Nazish Bostan

**Affiliations:** 1 Department of Biosciences, COMSATS University (CUI), Tarlai Kalan, Chak Shahzad, Islamabad, 45550 Pakistan; 2 Department of Paediatrics, Benazir Bhutto Hospital, Rawalpindi, Pakistan

**Keywords:** gastroenteritis, morbidity, Coinfection, mortality, disease burden

## Abstract

**Background:**

Acute diarrhea is a leading cause of morbidity and mortality in children particularly in developing countries of Asia and Africa. The present study was conducted to detect the two most important pathogens*, rotavirus* and *Campylobacter Jejuni* in children suffering with diarrhea in Rawalpindi and Islamabad, Pakistan in 2014. The clinical and epidemiological aspects of the disease were also investigated.

**Methods:**

A total of 500 stool samples were collected from children presented with clinical signs and symptoms of acute diarrhea. The samples were initially screened for the presence of rotavirus A (*RVA*) via ELISA (Enzyme-linked immunosorbent assay) and RT-PCR (Reverse Transcriptase PCR) and then were analysed for *C. jejuni* by using species specific PCR assay.

**Results:**

The detection rate of *RVA* was 26.4% (132/500) while, *Campylobacter* was detected in 52% (260/500) of samples with *C. jejuni* accounted for 48.2% (241/500) of all study cases. Co-infection of *C. jejuni* with *RVA* was identified in 21.8% of all cases. Children with *RVA* and *C. jejuni* co-infection showed a higher probability (*p* = 0.01) to be dehydrated. A significant association (*p* = 0.02) was found between *C. jejuni* positive status and fever in children. The median age of children with both *RVA* and *C. jejuni* infection was 6–11 months. The *RVA* detection rate was high in winter months of the year while, *C. jejuni* infections were documented high in summer over 1 year study period.

**Conclusions:**

The overall results have demonstrated the high prevalence of *C. jejuni* in Rawalpindi, Islamabad, Pakistan in 2014. The results of present study will not only help to calculate disease burden caused by *C. jejuni* and rotavirus but also will provide critical information to health authorities in planning public health care strategies against these pathogens.

## Background

Childhood diarrhea is defined as the passage of three or more abnormally watery stools within 24 h [1]. Globally, diarrheal diseases ranked as the second leading cause of death in infants and young children, contributed 2.5 million deaths annually [2]. The developing countries have the highest disease burden caused by diarrhea with almost four fifths of all under five deaths occur in Sub-Saharan Africa and South Asia [3]. According to an estimate by WHO (World health organization) in 2016, the under-five mortality rate in low-income countries was 73.2 deaths per 1000 live births which in nearly 14 times the average rate in developed countries [4]. Diverse parasitic, bacterial and viral agents are involved in diarrheal disease [5, 6]. Although, every diarrheal pathogen can cause disease alone, but 2 or more pathogens can also be responsible for the incidence of diarrhea, referred to as co-infections [7, 8]. Infections caused by *RVA* and *Campylobacter* can be of varying degrees from very mild to very severe resulting in complications [9].

Campylobacter is one of the four common causes of bacterial gastroenteritis both in developed and developing countries [10]. *Campylobacter* species are fastidious Gram negative, non-spore forming bacteria [11]. *Campylobacter jejuni* is the most prevalent species of genus *Campylobacter being major cause of bacterial gastroenteritis worldwide* [12]*.* The clinical symptoms range from moderate watery diarrhea to severe inflammatory diarrhea which may lead to complications including Guillain Barre’ Syndrome [13]*.* In developing countries *C. jejuni* is responsible for 0.4 episode of diarrhea per child per year [14].

Group A rotaviruses (*RVAs*) are considered as the leading cause of fatal dehydrating diarrhea in infants and young children causing 215, 000 deaths worldwide [15–17]. The genus *Rotavirus* (family *Reoviridae*; sub family *Sedoreovirinae*) is classified into nine recognized species (*RVA-RVI*) and another proposed species (*RVJ*) was identified recently in bats in Serbia [18]. In humans rotavirus strains belong to group A are major cause of disease [19]. To date, 36 G and 51 P genotypes have been reported worldwide [20]. The aetiology of *RVA* diarrhea is well understood globally with other pathogens [21].

Pakistan is one of the five countries with highest morbidity and mortality associated with diarrhea [22]. According to CDC (Centre of disease control) diarrhea is the second leading cause of death in Pakistan [23]. Regrettably, there is a lack of proper health facilities, continuous monitoring programs, proper trainings and advanced research laboratories facilities in Pakistan. The highly populated areas of Pakistan are suffering from poor water quality and bad sanitary conditions [24]. In view of all these circumstances, diarrhoea remains a major public health problem in Pakistani population.

The present study was designed to access and compare the prevalence of rotavirus and Campylobacter Jejuni among children with diarrhoea admitted in two major hospitals of Rawalpindi and Islamabad, Pakistan. The findings of this study will add to the available data on *RVA* and *C. jejuni* associated disease burden and epidemiology in Pakistan. Furthermore it will provide critical information to health experts and researchers in planning public health care strategies and will make them consider effect of co-infections in designing antimicrobial drugs in future.

## Methods

### Consents of study participants

Written permission were taken from the parents/guardians of study participants (children). Ethical approval was taken from the respective ethical committees of Pakistan Institute of Medical Sciences (PIMS), Benazir Bhutto Shaheed Hospital (BBH) and Internal Review Board (IRB) of COMSATS Institute of Information Technology, Islamabad.

### Study sites

Rawalpindi and Islamabad are the third most populous metropolitan cities of Pakistan. The high population size (4.5 million) strengthen the epidemiological monitoring of infectious diseases including gastroenteritis. Benazir Bhutto Shaheed Hospital Rawalpindi (BBH) is a public sector tertiary care hospital with heavy influx (2500 daily) of patients visiting hospital in OPDs (Out patient departments). The hospital is located on the main road in the crowded population of the city. Pakistan Institute of Medical Sciences (PIMS) is research oriented health sciences institute. This is a leading institute for training of doctors and other health staff from all over Pakistan. It is the major referral tertiary care hospital of Capital city Islamabad. There are 200 hospitalizations with 9000 cases in OPD/day in this hospital. The main target of the hospital is to provide health facilities not only to the residents of Rawalpindi/Islamabad but to the people of Northern Areas, Azad Jammu and Kashmir, NWFP (*North-West Frontier Province)* and Northern areas of Punjab.

### Study design

This sentinel surveillance study included 500 children of less than 5 years of age hospitalized or received treatment for acute diarrhea in the emergency paediatric ward of two hospitals, BBH (Rawalpindi) and PIMS (Islamabad), Pakistan. The Performa for the present study was designed to record the demographical and clinical characteristics of patients including patients day of onset of diarrhoea, date of admission in hospital, date of the collection of stool samples, body temperature (°C or °F), dehydration status, duration and episodes of the diarrhea per day, episode and duration of the vomiting per day, gender, age (months), weight (kg), height (cm) and residence of the patients.

### Sample collection method

Total 500 stool samples were collected between January 2014 to December 2014 in stool collection vials from patients experienced more than 3 watery loose stools in the last 24 h, with illness duration less than 2 weeks. The children were enrolled between 9:00 AM to 2:00 PM from Friday to Saturday in year 2014. Faecal sample were collected from children with acute gastroenteritis in a 30 ml polystyrene faecal container with spoon (Dynarex) and were stored initially in Microbiology and Public health laboratory COMSATS, Islamabad at − 80 °C until further analysis.

### Isolation and identification of *Campylobacter jejuni*

Stool sample was directly streaked onto modified Charcoal Cefoperazone Deoxycholate Agar (CCDA) (Oxoid, Hampshire, England) containing CAT antibiotic supplement (Cefoperazone 8 mg/litter, Amphotericin B 20 mg/litter, Teicoplanin 8 mg/litter) (Oxoid, Hampshire, England) These plates were incubated under microaerophilic conditions (Oxoid Campygen sachets Oxoid, Hampshire) for 48 to 72 h at 42 °C [25]. The isolated colonies were primarily identified on the basis of Gram staining, catalase, hippurate hydrolysis and oxidase activity. For molecular identification, DNA extraction was performed using phenol/chloroform method which was a modified version of Cheng and Jiang [26]. A negative extraction control with PBS and positive control was included in the extraction as well as in each PCR runs in each batch. Species specific primer for the detection of *C. jejuni* are HipO-F, (GACTTCGTGCAGATATGGATGCTT) and HipO-R, (GCTATAACTATCCGAAGAAGCCATCA) were used [27]. Thermo cycler conditions were 95 °C for 5 min, followed by 35 cycles of 95 °C for 30 s, 52 °C for 45 s and 72 °C for 60s, and finally 72 °C for 10 min. Cj255 was used as positive control [27].

### Detection of group a Rotavirus in Faecal samples

The presence of group A rotavirus was initially determined by screening of prepared stool dilutions using commercially available enzyme immunoassay ProSpect™ test. The test was carried out according to manufacturer’s instructions.

### RT-PCR for VP7 and VP4 genes

RT-PCR was carried out for VP7 and VP4 gene fragments of 1062 and 876 bp respectively by using consensus primers (Beg9, End9 for VP7; VP41-17F, Con2 for VP4). The primer sequences used are Beg9 (5’GGC TTT AAA AGA GAG AAT TTC CGT CTG G3’), End9 (5’GGT CAC ATC ATA CAA TTC TAA TCT AAG3’). VP4_1-17F (5’GGC TAT AAA ATG GCT TCG C3’) and con2 (5’ATT TCG GAC CAT TTA TAA CC3’) [28, 29]. The extracted RNA template was denatured for 2 min at 95 °C followed by reverse transcriptase PCR (RT-PCR) was carried out by using the Qiagen OneStep RT-PCR Kit (Qiagen/Westburg, The Nederland). The RT-PCR conditions involved initial reverse transcription at (30 min at 50 °C), polymerase activation at (95 °C for 15 min), 40 cycles of amplification (denaturation: 45 s at 94 °C; annealing (45 s at 45 °C for VP4 and 45 s at 50 °C for VP7), product extension (1 min at 72 °C) with final extension (10 min at 72 °C) [30]. The resulting PCR products were run on a polyacrylamide gel, stained with Ethidium Bromide (EtBr, Sigma Aldrich) and visualized under ultra violet light (UV-light).

### Statistical analysis

Statistical analyses were performed with SPSS software (v21.0) [31]. A Chi Square test was performed to test the possible difference between gender and *RVA* and *Campylobacter jejuni* status. For comparison of *RVA* and *C. jejuni* between different age groups, Student T test was performed. Descriptive statistics such as mean, median and standard deviation were calculated for other continuous variables like weight, height, fever, diarrhea duration and diarrhea episode, vomiting duration and vomiting episodes of the study. Statistical significance was defined as *p* < 0.05.

## Results

### Prevalence of *RVA* and campylobacter. Jejuni

**Fig. 1:**
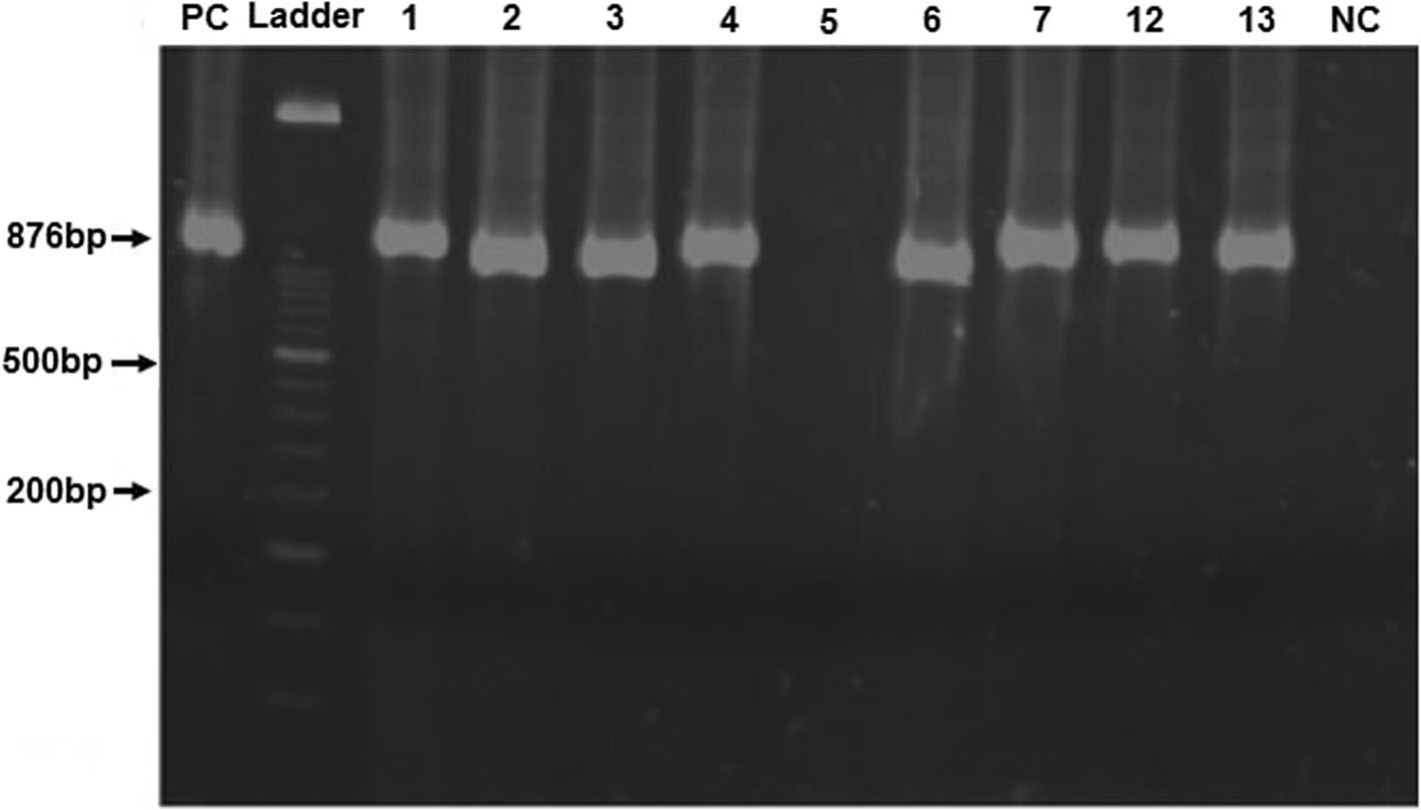
Identification of *rotavirus A* VP4 gene segment by using VP4 1-17F and Con2 primers: Lane 1: Positive control, Lane 2: DNA ladder 50 bp, lane 3, 4, 5, 6, 8, 9, 10, 11: Amplified product of gene segment VP4 (876 bp size) of samples no 1, 2, 3, 4, 6, 7, 12 and 13, Lane7: Samples number 5 negative for VP4 gene, Lane 12: Negative control

**Fig. 2:**
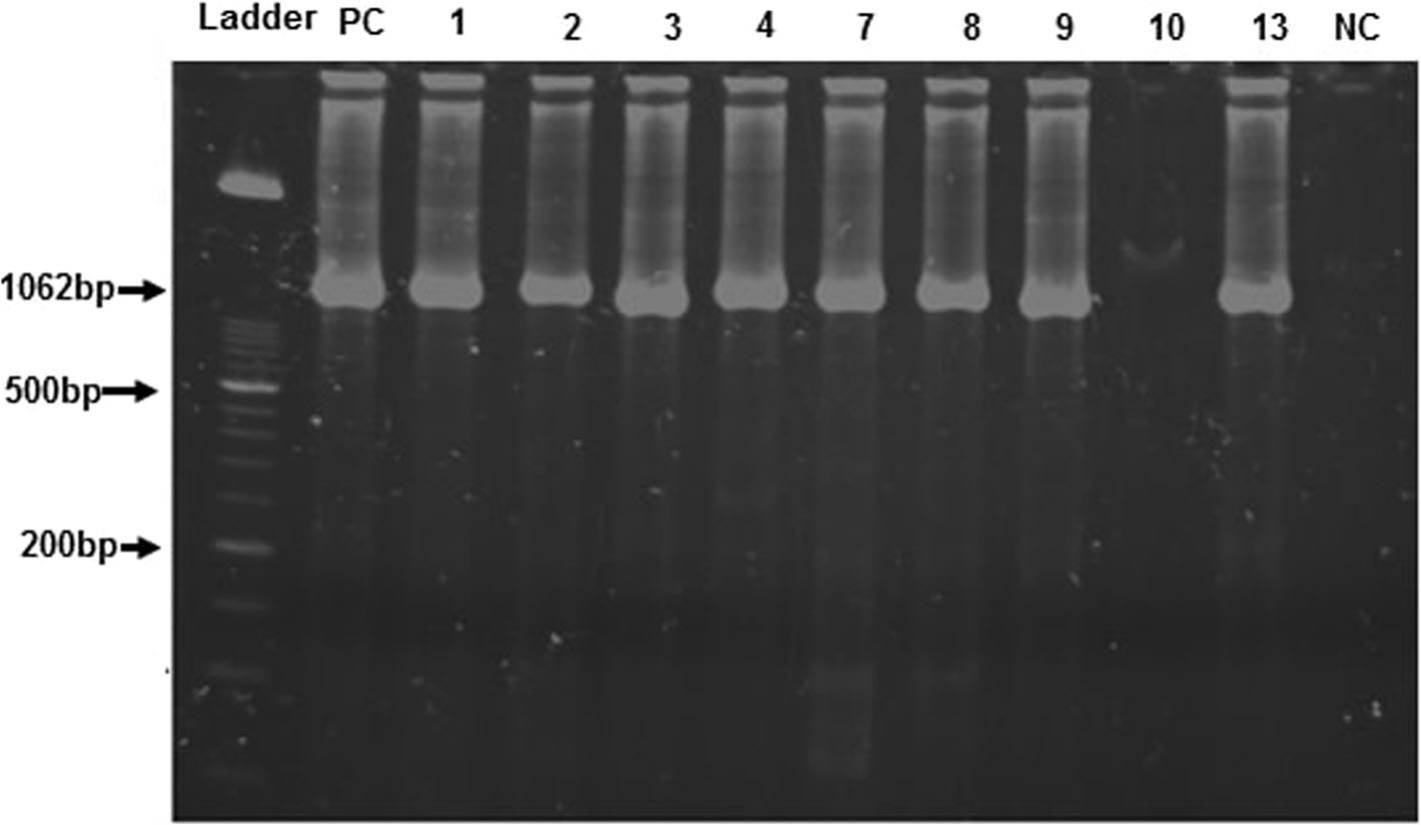
Identification of *rotavirus A* VP7 gene segment by using Beg9 and End 9 primers: Lane 1: DNA ladder 50 bp, Lane 2: positive control,:, lane 3, 4, 5, 6, 7, 8, 9, 11: Amplified product of gene segment VP7 (1062 bp size) of samples no 1, 2, 3, 4, 7, 8, 9 and 13, Lane 10: Samples number 10 negative for VP7 gene, Lane 12: Negative control

**Fig. 3:**
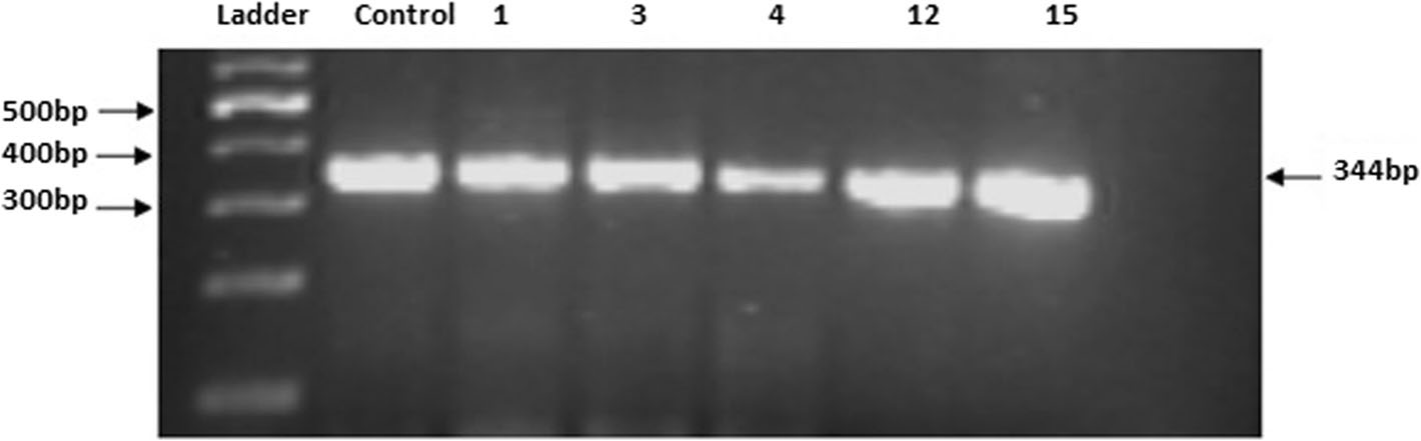
Identification of *Campylobacter jejuni* using HipO-F and HipO-R primers: lane 1 DNA ladder 100 bp, lane 2 positive control (cj 255), lane 3 to 7 human isolates [1, 3, 4, 12, 15] lane 8 negative control

A total of 500 stool samples from diarrheic children were analysed for the detection of rotavirus and *C. jejuni*. The ELISA for *RVA* was conducted for all stool samples and 132 samples were found to be positive through ELISA. Then, RT-PCR conducted for all ELISA positive samples showed 94.7% (125/132) of these samples were *RVA* positive by RT-PCR. The results of RT-PCR for *RVA* are shown in Figs. 1 and 2. *Campylobacter* was detected in 52% (260/500) of samples, with *C. jejuni* accounted for 48.2% (241/500) of all study cases. The results of PCR for *Campylobacter Jejuni* are shown in Fig. 3. The prevalence of rotavirus was found to be 26.4% (132/500) while, coinfection of *C. jejuni* and *RVA* was detected in 21.8% (109/500) of study samples. Total 236(47.2%) samples were found negative for both rotavirus and *C. jejuni*.

### Socio-demographic and clinical characteristics of study population

#### Rotavirus a

**Fig. 4:**
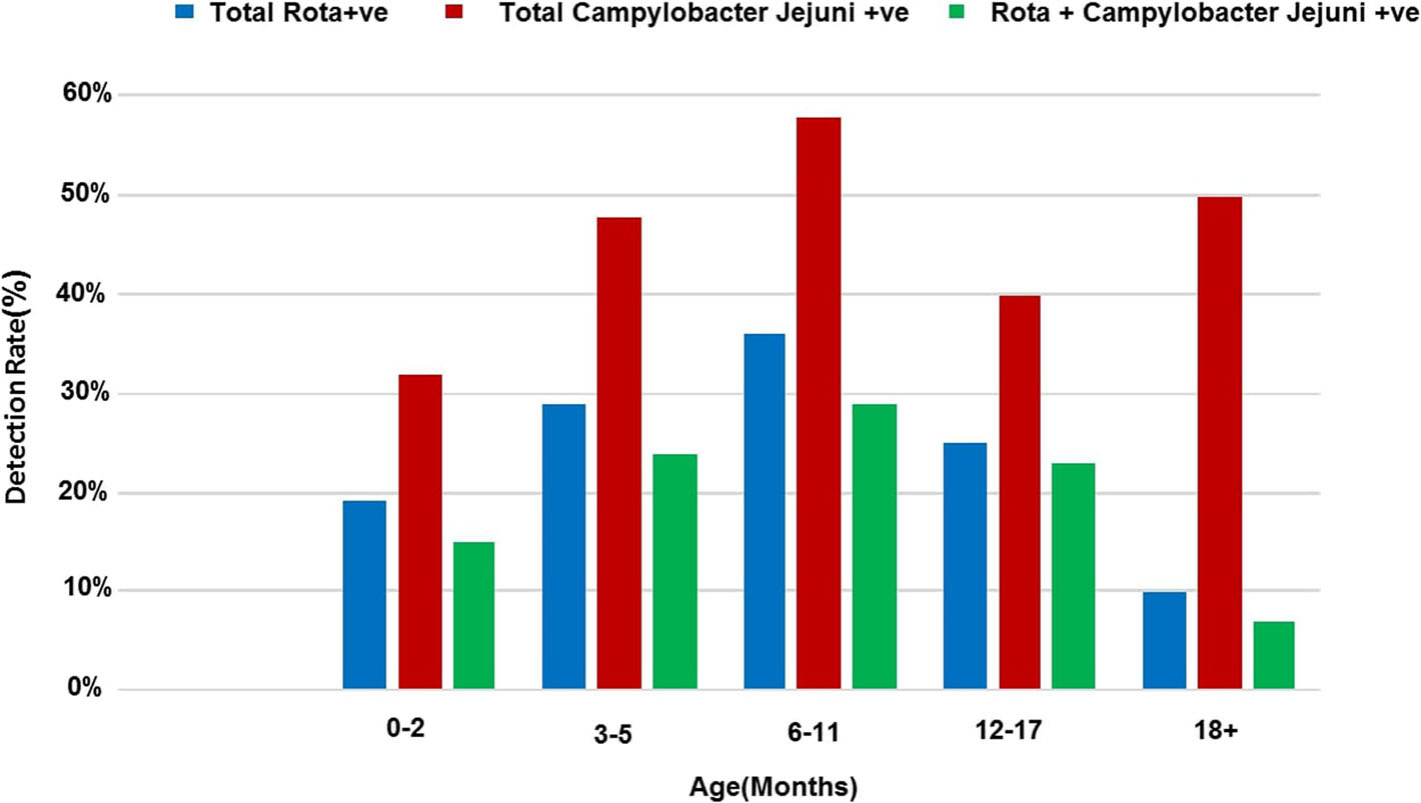
Age-wise Distribution of *rotavirus A s* and *Campylobacter Jejuni* infections among children with acute gastroenteritis (AGE) in Rawalpindi, Islamabad during year 2014

There was a statistically significant association found between gender and *RVA* gastroenteritis *(p* = 0.001). The incidence of *RVA* was higher in males than in females (Table 1). There was a statistically significance association found between mean age and *RVA* positive status (*p* = 0.03). However, according to the results of ELISA, highest rates of *RVA* infections were detected in children of 6–11 months of age and lowest in children > 18 months of age (Fig. 4). A statistically significant association was observed between dehydration and *RVA* gastroenteritis (*p*<0.009). However, other demographic and clinical characteristics (weight, height, temperature, diarrhoea duration and episodes, vomiting duration and episodes) had no statistically significant association with *RVA* gastroenteritis (Table 2).

**Table 1:** Gender wise distribution of *RVA* and *campylobacter Jejuni infections* among children with acute gastroenteritis (AGE) during year 2014

Rotavirus					
	RV (+)	RV (−)	Total	Chi-Square value	*p*-value
	(*n* = 132)	(*n* = 368)	(*n* = 500)	13.614	0.001*
Gender					
Male	99(32.1%)	209(67.9%)	308(61.6%)		
Female	33(17.2%)	159(82.8%)	192(38.4%)		
	*C. jejuni*				
	Campylo (+)	Campylo (−)	Total		
	(*n* = 241)	(*n* = 279)	(n = 500)	8.182	0.004*
Gender					
Male	164(53.2%)	164(53.2%)	308(61.6%)		
Female	77(40.1%)	115(59.9%)	192(38.4%)		
*C. jejuni* + Rota					
	Campylo+Rota (+)	Campylo+Rota (−)			
	(*n* = 109)	(*n* = 391)	(n = 500)	8.197	0.005*
Gender					
Male	80(26%)	228(74.02%)	308(61.6%)		
Female	29(15.1%)	163(84.9%)	192(38.4%)		

*P*<0.05 was considered statistically significant*

**Table 2:** Comparison of demographic and clinical features of children with *campylobacter Jejuni* and *RVA* positive gastroenteritis during year 2014

Rotavirus			
Demographic and Clinical Characteristics	Mean ± SD		*p*-value
	RV + ve (n = 132)	RV -ve (n = 368)	Total(n = 500)
Mean Age (in months)	11.02 ± 17.08	16.30 ± 26.31	0.03*
Weight (in Kg)	6.76 ± 3.39	7.38 ± 5.01	0.24
Height (in cm)	69.39 ± 11.72	71.36 ± 15.41	0.18
Temp (in C°)	28.63 ± 10.02	28.58 ± 10.20	0.26
Vomiting Duration (days)	2.65 ± 1.50	2.57 ± 1.80	0.65
Vomiting Episode/24 h	4.60 ± 4.52	4.40 ± 5.54	0.56
Diarrhea Duration (days)	2.77 ± 1.46	2.72 ± 1.74	0.80
Diarrhea Episode/24 h	19.48 ± 5.75	19.41 ± 6.35	0.91
Dehydration	0.63 ± 0.48	0.53 ± 0.50	0.009*
Campylobacter Jejuni			
Demographic and Clinical Characteristics	Mean ± SD		*p*-value
	Campylo +ve (n = 241)	Campylo -ve (*n* = 259)	Total(n = 500)
Mean Age (in months)	13.44 ± 20.18	16.31 ± 27.58	0.18
Weight (in Kg)	6.90 ± 3.70	7.52 ± 5.37	0.13
Height (in cm)	70.41 ± 12.73	71.31 ± 16.11	0.49
Temp (in C°)	37.78 ± 0.33	37.31 ± 0.39	0.02*
Vomiting Duration (days)	2.63 ± 1.62	2.55 ± 1.80	0.59
Vomiting Episode/24 h	6.58 ± 5.84	6.06 ± 5.27	0.29
Diarrhea Duration (days)	2.74 ± 1.60	2.72 ± 1.73	0.88
Diarrhea Episode/24 h	19.60 ± 6.29	19.31 ± 6.07	0.60
Dehydration	0.60 ± 0.49	0.51 ± 0.50	0.02*
RV+ Campylobacter Jejuni (Co-infection)			
Demographic and Clinical Characteristics	Mean ± SD		*p*-value
	Campylo+rota+ve (n = 109)	Campylo+rota-ve(n = 391)	Total(n = 500)
Mean Age (in months)	11.08 ± 16.77	15.97 ± 25.92	0.06
Weight (in Kg)	7.21 ± 2.82	7.36 ± 5.03	0.18
Height (in cm)	70.85 ± 11.23	71.14 ± 15.74	0.39
Temp (in C°)	37.77 ± 0.35	37.74 ± 1.94	0.42
Vomiting Duration (days)	2.60 ± 1.54	2.58 ± 1.77	0.69
Vomiting Episode/24 h	6.31 ± 5.68	6.27 ± 5.52	0.72
Diarrhea Duration (days)	2.73 ± 1.48	2.72 ± 1.72	0.69
Diarrhea Episode/24 h	19.38 ± 5.68	19.45 ± 6.40	0.90
Dehydration	0.63 ± 0.48	0.53 ± 0.50	0.01*

*P*<0.05 was considered statistically significant

#### Campylobacter jejuni

There was a statistically significant association observed between gender and *C. jejuni* infection (*p* = 0.004). The prevalence of *C. jejuni* was higher in males than in females (Table 1). There was no statistically significant relationship between age and *C. jejuni* positive status (*p* = 0.18) (Table 2). The detection rates of *C. jejuni* ranged between 32% among age 0–2 months to about 58% among age 6–11 months (Fig. 4). There was statistically significant association observed between dehydration and *C. jejuni* positive status *(p* = 0.02). Significant temperature difference was observed between cases of *C. jejuni* gastroenteritis and gastroenteritis due to other causes (*p* = 0.02) (Table 2). However, other demographic and clinical characteristics (weight, height, diarrhoea duration and episodes, vomiting duration and episodes) had no statistically significant association with *C. jejuni* gastroenteritis (*p* > 0.05) (Table 2).

#### *RVA* and *C. jejuni* co-infection

There was a significant association observed between gender and *RVA*-*C. jejuni* co-infection cases *(p* = 0.004) (Table 1). There was no statistically significant association found between age and *RVA*-*C. jejuni* co-infection cases (*p* = 0.06). There was significance difference observed between dehydration and *RVA-C. jejuni* co-infection status *(p* = 0.01). However, other demographic and clinical characteristics (weight, height, temperature, diarrhoea duration and episodes, vomiting duration and episodes) were statistically not significantly correlated with *RVA* and *C. jejuni* Co-infection cases (*p* > 0.05) (Table 2).

### Seasonality

**Fig. 5:**
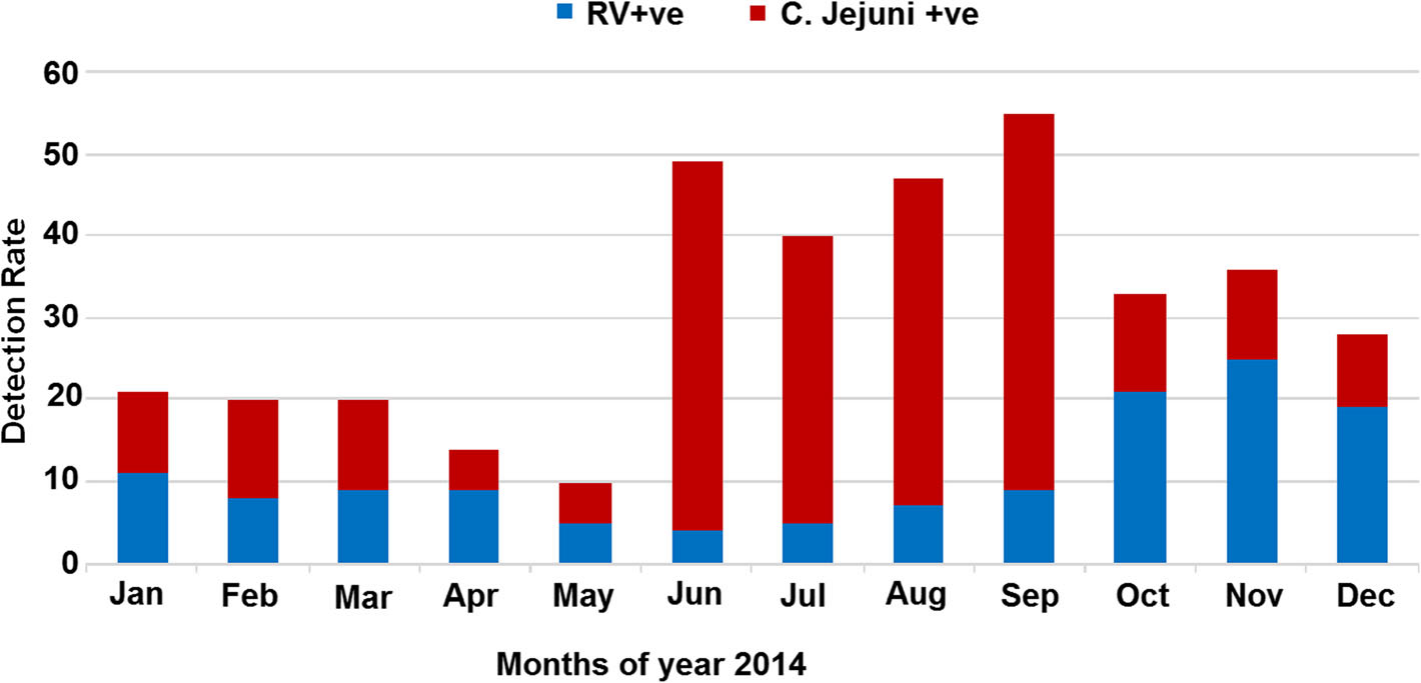
Month-wise Screening of human Group A *rotavirus* and *Campylobacter Jejuni* samples among children detected with acute gastroenteritis (AGE) in Rawalpindi, Islamabad during year 2014

The incidence of *RVA* and *C. jejuni* infections was observed throughout the year during 1 year of study period (January–December). However, the highest positive cases of *RVA* were detected in dry winter months (October to December) of the year 2014. The highest prevalence of *C. jejuni* infections occurred during summer months of the year 2014 (June to September) (Fig. 5).

## Discussion

Acute gastroenteritis continuous to be a serious health dilemma in both developed and developing countries [10]. According to a recent estimate 36,862 children die every year due to diarrhoea in Pakistan [32]. Pakistan is supported by WHO (World health organization) and its partner GAVI (Global Alliance for Vaccine and Immunization) to control communicable disease burden including diarrhea. However still there is poor state of health care system in Pakistan. Keeping in mind the importance of proper surveillance program in the country we have determined the prevalence of *rotavirus* and *Campylobacter Jejuni* in two major hospitals of Rawalpindi, Islamabad, Pakistan during 2014.

In this hospital-based study prevalence of *C. jejuni* was 48.2% while 26.4% children were infected with *Rotavirus A* (*RVA*). The incidence of co-infection was found to be 21.8% in all study samples. The *RVA* prevalence in the present study is quite similar to the previous reported rate (29–34%) in Pakistan [33–35]. *Campylobacter* contributed highest disease burden of diarrhea in Pakistan during year 2014 which is in accordance with the previous study conducted in Malawi [13]. The prevalence of *C. jejuni* detected in previous studies conducted in Military hospital in Rawalpindi, Islamabad, Pakistan and Agha Khan Hospital Karachi was lower than the present study [36, 37]. There might be several reasons for this low prevalence, such as a different study design and sampling period, type of health facilities and diagnostic procedures in target hospital and patients age and patient testing standards.

In the present study *RVA* infection was detected mostly in children of 6–11 months of age. These results are in accordance with previous studies from Pakistan showing majority of children effected with *RVA* infection are <2 years of age [33, 35, 38, 39] and other countries of the world [40–43]. The reason of high *RVA* infection in lower age may be due to low immunity in children of less age.

The prevalence of *C. jejuni* was found to be highest in the age group 6–11 months similar to *RVA*. However, the prevalence of *RVA* in children of > 18 months of age with diarrhea was 10% compared with 50% in *C. jejuni* in the current study.. *Campylobacter*, therefore contributed largely to diarrheal infection in children more than 12 months of age. There is a possibility of repeated exposures to *Campylobacter* species from different sources for the whole childhood period which may elucidating the high prevalence in children > 1 years of age [13]. The mortality rate due to diarrhea is decreasing with increasing age of children however morbidity remain constant in adult population [44].

*Rotavirus* A infections commonly present during the winter months in temperate climates. However, in most tropical areas *RVA* causes enteritis throughout the year without seasonal variation [45]. Pakistan is located in the temperate zone (between latitudes 25° and 36° N) with extreme temperature variations. In the present study *RVA* infection was present throughout the year with increasing frequency of *RVA* positive cases in winter. The results of present study are similar to previous studies reported in Pakistan with *RVA* predominance throughout the year [33–35, 38]. The same seasonal pattern was reported in Bangladesh, India and Thailand [46–48].

The seasonal pattern of *Campylobacter* infection varies from country to country as well as within a country. In developing countries, *Campylobacter* enteritis has no seasonal fluctuation while in developed countries its epidemic peaks are in summer and winter [49, 50]. In present study *Campylobacter* was detected throughout the year with prominent peaks from June to September which is consistent with previous studies from Pakistan and Malawi [13, 36, 51, 52].

There are variations in associations between co-infection and clinical characteristics of study population. Some studies have reported more severe diarrhoea in co-infection cases, while other studies have found no difference between mono-infection and co-infection [7, 53]. Significant association was found between dehydration and co-infection. However, there were no significant association in the severity of diarrhea, vomiting and fever among children with single infection and co-infection.

*RVA* had significant association with dehydration but not with diarrhea, vomiting and fever. *RVA* infection contributed more to dehydration in children than *Campylobacter*. A significant high fever was observed among children with *campylobacter* infection than *RVA*. In comparison to frequency of diarrhea and vomiting between *rotavirus* and *campylobacter* no significant association was found. The mean height in *Campylobacter* and *RVA* positive was seen marginally lower than negative cases. The clinical results of present study are consistent with previous findings from other countries of the world [7, 54].

The short study period, small number of samples and lack of multiple study sites are the major limitations of this study. Therefore, the continued surveillance of pathegens causing diarrhea is mandatory in the country to assess the disease burden which will further help in developing informed disease prevention strategies against these pathogens.

## Conclusions

In conclusion, this hospital-based study of children hospitalized with diarrhea in Pakistan suggests the high disease burden of *Campylobacter Jejuni* in association with *rotavirus A* infection. *RVA* vaccine is included recently in the EPI Program of Pakistan as recommended by WHO (World health organization). It is predicted that after the introduction of *rotavirus* vaccine the bacterial agent including *Campylobacter* could play a leading role in diarrheal diseases in future. It is empirically important to conduct more studies and improve the existing diagnostic methods into fast less time consuming techniques for rapid diagnosis. Conclusively, further exploration regarding the cost of illness due to unsafe drinking water in the country should allow the government to construct and implement inherently efficient policies and schemes in the future.

## Data Availability

The datasets used and/or analysed during the current study available from the corresponding author on reasonable request.

## Abbreviations

BBH: Benazir Bhutto hospital
CDC: Centre of disease control
CUI: COMSATS University, Islamabad
ELISA: Enzyme-linked immunosorbent assay
EPI: Expanded program on immunization
GAVI: Global Alliance for Vaccines and Immunisation
IRB: Internal review board
NWFP: *North-West Frontier Province*
OPD: Outpatient door
PIMS: Pakistan institute of medical sciences
RT-PCR: Reverse Transcriptase PCR
SPSS: Statistical package for the social sciences
WHO: World health organization
